# Impact of fruits and vegetables vouchers on food insecurity in disadvantaged families from a Paris suburb

**DOI:** 10.1186/s40795-019-0289-4

**Published:** 2019-04-04

**Authors:** Camille Buscail, Judith Gendreau, Paul Daval, Pierre Lombrail, Serge Hercberg, Paule Latino-Martel, Chantal Julia

**Affiliations:** 1 Equipe de Recherche en Epidémiologie Nutritionnelle (EREN), Université Paris 13, Inserm U1153, Inra U1125, Cnam, Centre de Recherche en Epidémiologie et Biostatistiques (CRESS) Sorbonne Paris Cité, Bâtiment SMBH -74 rue Marcel Cachin, 93017 Bobigny cedex, France; 2 Département de Santé Publique, Hôpital Avicenne (AP-HP), 125 rue de Stalingrad, 93000 Bobigny, France; 3 Maison de la Santé de Saint-Denis, 6 rue des Boucheries, 93200 Saint-Denis, France; 4 Laboratoire Educations et Pratiques de Santé, Campus Condorcet, Université Paris, 13, 74 rue Marcel Cachin, Bobigny, France

**Keywords:** Food insecurity, Food vouchers, Community-based participatory research

## Abstract

**Background:**

Social inequalities in nutrition lead a high number of families to struggle with food insecurity, even in developed countries. We aimed to assess the impact of fruits and vegetables vouchers on food security among disadvantaged households from a Paris suburb.

**Methods:**

We used a pre-post assessment design. Families answered face-to-face questionnaires on food consumption and food security status before and after a randomly assigned intervention. Households in the intervention group received vouchers to buy exclusively fruits and vegetables over one year. Both intervention and control groups benefitted from nutritional education through workshops performed by dieticians during the study period. The Household Food Security Module (HFSM) was used to assess food security status of households at inclusion. Food Insufficiency Indicator (FSI) was used to assess food security at inclusion and follow-up. Evolution of FSI on both groups was evaluated using McNemar test.

**Results:**

Among the 91 families included between May 2015 and May 2016, 64 completed the post assessment questionnaire. At inclusion, 68.3% of families were experiencing food insecurity and 78.1% were experiencing food insufficiency. No association was found between food consumptions and food security status. After one-year follow-up, the prevalence of food insufficiency was significantly decreased in the intervention group (61.8%, with *p* value = 0.03), and unchanged in the control group.

**Conclusion:**

In this pilot study, food insufficiency was significantly decreased in families receiving vouchers for fruits and vegetables over a one-year period.

**Trial registration:**

NCT02461238, registered 3 June 2015 – Retrospectively registered, https://clinicaltrials.gov/ct2/show/NCT02461238

## Background

The Food and Agriculture Organization (FAO), stated that food security exists “when people at all times, have physical, social and economic access to sufficient, safe and nutritious food that meets their dietary needs and food preferences for an active and healthy life” [1]. Conversely, food insecurity (FI) occurs when people cannot have access to food according to the criteria defined above [1, 2]. FI is associated with low diet quality and adverse health effects in both adults and children [3–11]. In France, like in many developed countries, disadvantaged families are more likely to experience FI [12–16]. According to a study performed in the French general population in 2016, 11% of adults and 12% of children had experienced FI over the last 12 months [17], and these prevalence were increased compared to those of a similar study performed 10 years before [12, 13]. Despite a considerable web of food aid structures – more than 250 were identified during a study performed in France in 2012 among the beneficiaries of food aid – a large number of disadvantaged households are exposed to FI [18–20]. Besides, FI is not entirely explained by financial constraints [12, 21].

In line with previous studies, the INCA2 study performed among the French general population showed that surveyed households with FI were mainly made of women and/or young people, facing barriers other than money to reach food security [13]. This indicator seems therefore relevant to be assessed beyond usual poverty markers.

Several studies assessing the impact of specific food vouchers on nutritional behaviour among disadvantaged population have been conducted in Europe, USA, and New-Zealand [22–28]. Overall, they show positive results on dietary habits, particularly when supported by a nutritional education program. In the USA, the main current nutritional support programs (including WIC, SNAP and CEP) have shown their effectiveness in reducing the prevalence of FI [29–34]. In France, to the best of our knowledge, no work has evaluated the impact of a nutritional support program on food insecurity. The pilot interventional study “*Fruits et légumes à la Maison*” (FLAM) was conducted in a Paris suburban city. It primarily aimed to assess the impact of fruits and vegetables vouchers and nutritional education over a one-year period on fruits and vegetables consumption among children from low-income families. The purposes of this ancillary analysis of the FLAM study were to 1) describe food insecurity prevalence and its association with sociodemographic characteristics and food consumption, and 2) determine whether the intervention improved food security among FLAM participants.

## Methods

### Study design

**Fig. 1:**
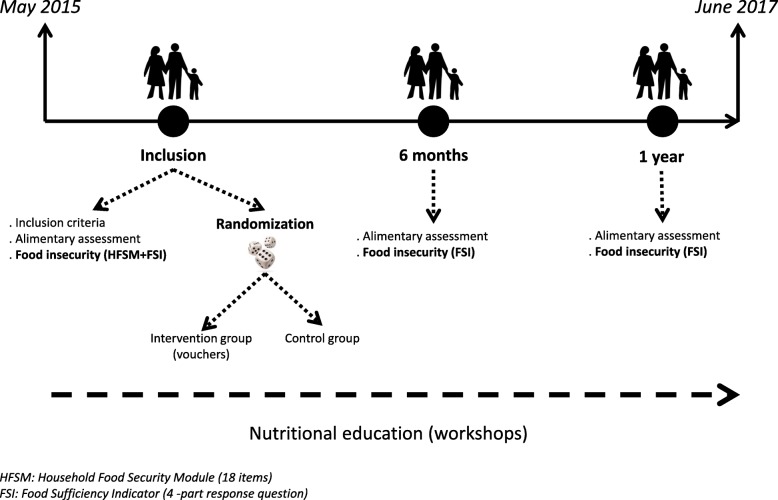
General description of the FLAM study

The study design has been fully described elsewhere [35]. Briefly, we used a pre-test - post-test design for this interventional study, and participants were randomized in a control group or in an intervention group at inclusion (Fig. 1). The study took place in Saint-Denis (Seine-Saint-Denis county, Ile-de-France region, France), a disadvantaged suburb city of Paris. The intervention group received at home, during one-year period, vouchers allowing exclusively the purchase of fruits and vegetables (including fresh, canned or frozen vegetables and 100% fruit juices). The amount of the vouchers was proportional to the size of the household, ranging from 12 € per month for single-parent families with one child, to 24 € per month for households made of at least 4 persons. In parallel, each group were proposed to participate to nutritional and culinary workshops performed by dieticians in their neighbourhood.

### Inclusion criteria

Families with at least one child aged from 3 to 10 years old, living in the northern districts of Saint Denis were eligible to the study. For families with several children matching with the age criterion, we included the child with the closest anniversary birth date (regarding the inclusion date). In addition, adult participants had to be unemployed, or benefit from social minima (Active Solidarity Income, Allocation of minimum pension) or any income-terms allowance, or have incomes below the poverty line. The poverty line threshold was defined using the French National Statistical Institute (*INSEE*) according to the French incomes data (that is, 1234€ per month rounded up to 1300€ for a single-parent household with at least one child aged under 14 years old, and 1777€ per month rounded to 2000€ for a couple with at least one child aged under 14 years old) [36, 37]. Finally, French language had to be well spoken and understood by participants.

### Data collection

Data were collected via face-to-face questionnaires administered by trained interviewers at inclusion, after 6 months and one year. Volunteer families were interviewed at community centres, or at home in order to sign the consent form, and complete the questionnaires. Questionnaires were adapted from those used in the ABENA study, which was specifically designed to be administered to disadvantaged groups [20]. A food frequency questionnaire was used to describe the consumption of children and adults in 13 main food groups (cereal products, starches, vegetables, fruits, legumes, dairy products, meats and eggs, fish and sea-food products, fast-food and pizza, salty snacks, sweet products, and beverages). The baseline questionnaire also included information on inclusion criteria, living conditions, and food security (see details below). We relied upon the latter data and sociodemographic characteristics to compute the EPICES score [38]. Based on 11 questions on various socioeconomic determinants, this individual score assesses the precariousness level of subjects living conditions. It ranges from 0 (the less precarious situation) to 100 (the most precarious situation), with a threshold of 30.17 to define precariousness, a score upper than 53.84 reflecting a great precariousness. The baseline questionnaire had a duration of about one hour, and follow-up questionnaires about 30 min. The vouchers were electronically traceable, so we were able to know exactly the number of vouchers used for each household. After the interview, parent-child pairs were assigned in the intervention or in the control group through an algorithm of random distribution performed on a laptop. The algorithm was computed to balance groups every 50 inclusions.

### Ethics, consent and permissions

Each adult participant (whether the mother or the father included with his/her child) signed a consent form, after the interviewer made sure it was well understood. The study was approved by the Ethics Review Committee of the National Institute of Health and Medical Research (*Institut National de la Santé et de la Recherche Médicale*) (Inserm) IRB00003888 under the number 15–247. The declaration to the National Commission of Data Processing and Liberties (*Commission Nationale de l’Informatique et des Libertés*) (CNIL) of February 26, 2015 was made under number 1838429v0. The study protocol has been registered on clinical trials website under no. NCT02461238.

### Assessment of food insecurity

At inclusion, the 18-item Household Food Security Module (USDA HFSM), validated by the US Department of Agriculture (USDA), was used to measure food security [39]. We used the French version of the questionnaire, adapted from the first translation performed in Quebec for the 2004 cycle of the Canadian Community Health Survey [40, 41], that was already used in two French studies ABENA [5, 20] and SIRS [14]. Based on the 18 items asked in order of severity, we used the guide provided by Bickel and colleagues to compute a continuous measure (ranging from 0 to 10) of FI, which was divided according the usual thresholds to obtain the 3 following categories: a) food secure (0–2.32), b) food insecure, without hunger (2.33–4.55), c) food secure with hunger (> 4.55) [42]. We also used the USDA Food Sufficiency Indicator (FSI), a single question with a four-part response [43]. This measure allows scaling FI in four categories: a) food security, b) qualitative food insecurity (“enough but not the kinds we want combined”), c) quantitative food insecurity sometimes (“sometimes not enough”, and d) frequent quantitative food insecurity (“often not enough”). Given the heaviness of the 18-items module, and to shorten interview time, follow-up assessments of FI, were done only using the FSI. We relied on a comparative analysis showing that FSI was fairly accurate to estimate FI when combining the three categories of food insecurity described above compared to module with more questions [43]. Given the small number of participants, we combined the two grades of the FSI that was therefore assessed through one two-category variable: food sufficiency and food insufficiency (including quantitative and qualitative food insufficiency).

### Statistical analyses

The sample size calculation was based on the primary outcome of the FLAM study, which was defined by the proportion of low fruits and vegetables consumers (less than 3.5 servings of FV per day) in children. Based on the literature, the baseline proportion of low consumers was expected to be at 83.9%, and the target proportion of low consumers was expected equalling those of the French general population, 61.0% [5, 44]. This led to an expected number of participants of 92 in each group, leading to a total of 184 participants [35]. The percentage of people lost to follow-up was estimated to be about 40%, and the calculation took into account a type I error of 5% and with an expected power of 90%, leading to an expected number of participants of 300. Despite a wide range of recruitment strategies [45], we finally included 92 parent-child pairs, including 47 in the control group and 45 in the intervention group.

Comparison of sociodemographic characteristics according to the FI status were performed using Chi-square tests (or exact Fisher tests) for qualitative variables, and using Anova tests for quantitative variables. Dietary consumptions were described depending on the compliance to the French nutritional recommendations within each food group. They were compared according to FI status using Chi-square or Fisher exact tests. A Spearman correlation test between the USDA HFSM and the FSI was performed. Comparison of food insecurity between inclusion and one-year follow-up based on USDA FSI as a dichotomous variable (food secure vs. the 3 others categories) was performed using a McNemar test. We also performed a difference-in-difference assessing the evolution of the food insufficiency in the two study groups between inclusion and follow-up [46].

All statistical analyses were performed using SAS software (*version 9.4, SAS institute, Cary, NC, USA*).

## Results

**Fig. 2:**
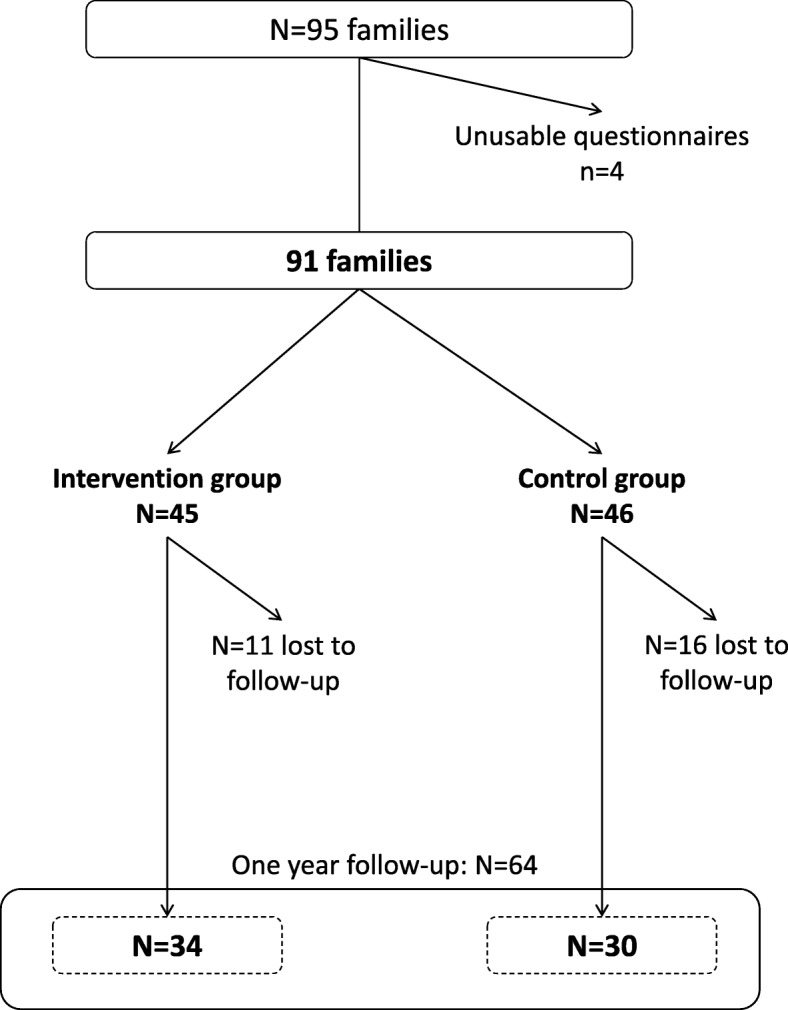
Flowchart of the study

Of the 95 families recruited from May 2015 to May 2016, 91 were finally included (Fig. 2). According to the USDA HFSM, 68.1% of households were experiencing FI, and more than a half of them (*n* = 38) were describing a FI with hunger. The overall proportion of FI was rising up to 80.2% when using the FSI. Qualitative FI was experienced by 47% of families and quantitative FI by 31% of families at inclusion. The USDA HFSM and FSI were positively correlated (Spearman correlation 0.59, with *p* < 0.0001). FI was significantly more frequent in single parent families (*p* = 0.01), when the financial situation of the household was perceived as difficult (*p* < 0.01), and when the deprivation EPICES score was higher (*p* < 0.001) (Table 1). No significant association was found between food consumptions and food insecurity (according to the USDA HFSM) at inclusion (Table 2). Among the 64 families who answered the last questionnaire after one-year follow-up, food insufficiency assessed by the FSI was significantly decreased in the intervention group (61.8% vs 85.3% at inclusion, with *p* = 0.03), while it remained stable (70%) in the control group (*p* = 1.0). However, the difference-in-difference test was not significant (*p* value = 0.12). No significant evolution of the food sufficiency status was observed depending on the participation to the nutritional workshops (Table 3). Overall 80.2% of vouchers were used, with similar proportion of vouchers used regardless the food sufficiency status after one year follow-up (data not tabulated).

**Table 1:** Sociodemographic characteristics of the population according to USDA Household Food Security Module (*N* = 91)

Food insecurity status (HFSM)	Food secure *N* = 29 (31.9%)		Food insecure, without hunger *N* = 24 (26.4%)		Food insecure, with hunger *N* = 38 (41.7%)		*p* value*
	mean	+/−SD	mean	+/−SD	mean	+/−SD	
Parent’s age (years)	39.4	7.9	40	7.2	39.6	7.2	0.81
Child’s age (years)	7.5	2.4	7.9	2	7.2	2.6	0.47
EPICES score	50.6	15	55.1	17.7	65	14.8	< 0.001
Budget for food (in € /individual/month)	89.6	49.5	90.4	25.3	108.8	63.9	0.13
Budget for FV (in € /individual /month)	25.0	17.5	28.6	17.5	30.6	21.1	0.26
	*n*	%	*n*	%	*n*	%	
Place of birth of the parent							
France	12	41.4	6	25	11	28.9	0.39
Other country	17	58.6	18	75	27	71.1	
Marital status							
Single	23	79	24	100	37	97.4	0.01
Cohabiting	6	21	0	0	1	2.6	
Size of the household							
2 people	8	27.6	4	16.7	9	23.7	0.93
3 people	7	24.1	7	29.2	10	26.3	
4 people and more	14	48.3	13	54.2	19	50	
Education level							
Primary school	6	20.7	7	29.2	16	42.1	0.10
Secondary school	10	34.5	9	37.5	15	39.5	
Baccalaureat and university	13	44.8	6	25	6	15.8	
Other	0	0	2	8.3	1	2.6	
Professional status							
Working	10	34.5	6	25	10	26.3	0.69
Unemployed	19	65.5	18	75	28	73.7	
Income level (in € per c.u.)							
260 € at the most	4	13.8	6	26.1	12	31.6	0.34
400 € at the most	13	44.8	10	43.5	18	47.4	
800 € at the most	12	41.4	7	30.4	8	21.0	
Perception of the financial situation of the household							
It’s OK	2	6.9	0	0	0	0	< 0.01
Need to be very cautious	15	51.7	10	41.7	6	15.8	
It’s difficult	8	27.6	9	37.5	16	42.1	
Often making debts	4	13.8	5	20.8	16	42.1	
Food aid use over the past 12 months							
No	22	78.6	19	79.2	26	70.3	0.65
Yes	6	21.4	5	20.8	11	29.7	
Purchasing frequency for FV							
Several times a week	12	41.4	9	37.5	18	47.4	0.32
Once a week	9	31	13	54.2	14	36.8	
2 or 3 times a month	8	27.6	2	8.3	5	13.2	
Study group							
Intervention	12	41.4	11	45.8	22	57.9	0.37
Control	17	58.6	13	54.2	16	42.1	

Abbreviations: *c.u* consumer unit, *€* Euros, *FV* Fruits and vegetables, *HFSM* Household Food Security Module, *SD* Standard Deviation

*Chi-square tests and exact Fisher tests were performed for qualitative variables and Anova tests were performed for quantitative variables;

Missing data: Budget for food and budget for FV: N = 3 (3.2%); Use of food aid N = 2 (2.2%); Income level and purchasing frequency for FV: *N* = 1 (1.0%);

**Table 2:** Food groups consumption according to food insecurity status at inclusion (N = 91)

USDA food insecurity status	Food secure *N* = 29		Food insecure, without hunger *N* = 24		Food insecure, with hunger *N* = 38		*p* value*
	*n*	%	*n*	%	*n*	%	
CHILDREN							
Fruits and Vegetables							
< 3.5 a day	19	65.5	14	58.3	26	68.4	0.73
≥ 3.5 a day	10	34.5	10	41.7	12	34.6	
Bread, starches and cereal products							
< 3 times a day	14	48.3	5	20.8	17	44.7	0.09
≥ 3 times a day	15	51.7	19	79.2	21	55.3	
Meat, poultry and fish							
1 to 2 times a day	12	41.4	10	41.7	10	27.8	0.42
Less than or more than 2 per day	17	58.6	14	58.3	26	72.2	
Dairy products							
3–4 per day	18	62.1	16	66.7	24	63.2	0.96
More or less than 3–4 per day	11	37.9	8	33.3	14	36.8	
Sugared products							
4 to 6 times a week at the most	8	27.6	5	20.8	13	34.2	0.56
One time a day or more	21	72.4	19	79.2	25	65.8	
Fatty and salty products							
2 to 3 times a week at the most	15	51.7	14	58.3	14	36.8	0.21
4 times a week and more	14	48.3	10	41.7	24	63.2	
Drinks							
Only or mainly water	22	75.9	20	83.3	24	63.2	0.22
Only or mainly other drinks than water	7	24.1	4	16.7	14	36.8	
ADULTS							
Fruits and Vegetables							
< 3.5 a day	25	86.2	15	62.5	31	81.6	0.09
≥ 3.5 a day	4	13.8	9	37.5	7	18.4	
Bread, starches and cereal products							
< 3 times a day	20	69	12	50	25	65.8	0.32
≥ 3 times a day	9	31	12	50	13	34.2	
Meat, poultry and fish							
1 to 2 times a day	12	41.4	12	50	13	35.1	0.50
Less than or more than 2 per day	17	58.6	12	50	24	64.9	
Dairy products							
3 per day	4	14.3	4	16.7	4	10.5	0.77
More or less than 3 per day	24	85.7	20	83.3	34	89.5	
Sugared products							
4 to 6 times a week at the most	19	65.5	16	66.7	25	34.2	1.0
One time a day or more	10	34.5	8	33.3	13	65.8	
Fatty and salty products							
2 to 3 times a week at the most	15	51.7	14	58.3	15	39.5	0.32
4 times a week and more	14	48.3	10	41.7	23	60.5	
Drinks							
Only or mainly water	20	69	22	91.7	28	73.7	0.11
Only or mainly other drinks than water	9	31	2	8.3	10	26.3	

* Chi-square or Fisher exact tests were performed

**Table 3:** Comparison of food insecurity status between baseline and one-year follow-up, using Food Sufficiency Indicator (FSI) (*N* = 64)

	Baseline		One year follow-up		*p* value*
	*n*	%	*n*	%	
All (*n* = 64)^†^					
Food sufficiency	14	21.9	22	34.4	0.10
Food insufficiency	50	78.1	42	65.6	
Intervention group (*N* = 34)					
Food sufficiency	5	14.7	13	38.2	**0.03**
Food insufficiency	29	85.3	21	61.8	
Control group (*N* = 30)					
Food sufficiency	9	30	9	30	1.00
Food insufficiency	21	70	21	70	
Participation in workshops (*N* = 42)					
Food sufficiency	7	16.7	13	30.9	0.11
Food insufficiency	35	83.3	29	69.1	
No participation (*N* = 22)					
Food sufficiency	7	31.8	9	40.9	0.44
Food insufficiency	15	68.2	13	52.1	

*Proportion of families with food insufficiency between inclusion and follow-up were compared within each group using McNemar tests

† Comparison of FSI between intervention and control groups at inclusion (Fisher exact test): *p* value = 0.23

Difference-in-difference test *p* value = 0.12

## Discussion

Results of this pilot study suggest that food insufficiency could be partly decreased in low-income households receiving vouchers for fruits and vegetables over a one-year period.

The prevalence of FI at inclusion in our study population was similar to that measured in the ABENA study, conducted among food aid users in France [20]. Qualitative FI was experienced by 43% of households in the ABENA study vs. 47% in the FLAM study, and quantitative FI was experienced by 31% of families in both FLAM and ABENA studies. Yet, only a small proportion of participants in FLAM reported having used food aid over the last 12 months (24%). Indeed, the study appeared to have reached families which did not benefit from food aid at the time of the study, though they were facing difficulties to reach food security. This underlies the need for increasing the knowledge on households experiencing FI, and the levers for reducing it at different levels. Such policies are relying upon the concept of “proportionate universalism” proposed by Michael Marmot in 2010 [47]. Based on the assumption that focusing solely on the most disadvantaged is not sufficient to reduce health inequalities, the author explains that actions must be universal, but with a scale and intensity that is proportionate to the level of disadvantage to reduce the steepness of the social gradient in health.

In France, the National Nutrition and Health Program (programme national nutrition santé, PNNS) is a national public health program aiming to improve the health of the general population through nutrition. Reducing nutritional health inequalities has become one of its priorities over the past few years, including use of vouchers for increasing FV consumption among disadvantaged households [48, 49]. Accessing to adequate nutritious food is a basic necessity, and therefore a basic human right [1, 50–52]. Moreover long-term adverse effects of FI on health have been widely reported [7, 9, 11]. Nutritional aid programs supported by evidence-based interventions have emerged in developed countries aiming to tackle FI among disadvantaged households [53–55]. Thus, several studies have assessed the impact of financial incentive programs including food vouchers, dietary counselling and farmer’s market incentive on dietary consumption and nutritional status among low-income populations [22, 56]. Yet, only a few of these programs focused specifically on the impact on food security status of the households. In the USA, participants to the Women, Infants and Children (WIC) program was associated with an improvement of the FI status [57]. Gundersen and colleagues showed a positive impact of the Supplemental Nutrition Assistance Program (SNAP) on the prevalence of FI in households with children by at least 6 percentage points [34]. In England, women recruited in the healthy Start program reported an improvement of the quality of family diets [23]. Targeting specific food groups of high nutritional quality like fruits and vegetables which are usually under-consumed in such populations seems efficient by guiding food choices, and therefore facilitating the shift of nutritional habits in the long term [7, 22, 23]. That being said, such intervention could be perceived as paternalistic, by interfering with individual autonomy or freedom of the targeted population [58–60]. It seems therefore important to keep in mind that from an ethical point of view, the priority always remains the decrease of the FI, with or without any impact on the overall nutritional quality. And despite using vouchers for purchasing FV (i.e. food items of high nutritional quality), we assume that results on food sufficiency could have been similar by using any kind of food vouchers.

Our results are in line with previous works suggesting that FI is not restricted to a money issue [12, 61]. First, the financial help we provided was modest, with a value limited to a maximum of 24 € per month for a 4 members household, whereas Anliker and colleagues suggested that a minimum allotment of 20 US$ per week was necessary [62]. More recently, a minimal allotment of 7.50 US$ per week was proposed by An and colleagues as suitable to entail an evolution of the purchasing behaviour [22]. Besides, a study performed in France among a similar population showed that about 20% of the households (called “positive deviants”) developed strategies allowing them to purchase healthier food than others, without increasing their food budget [63].

Not surprisingly, FI was increased when EPICES deprivation score was higher, when the perception of the household was more difficult, and among single-parent families [14]. Other associations between FI and parents’ education level, place of birth or income level were not significant. We did not highlight any significant association between FI status and dietary behaviours. However, this could be due to an insufficient power, given the low number of participants. Moreover, and given its inclusion criteria, our study population might have homogeneous dietary behaviour, which cannot be discriminated by FI status. The one-year duration of the intervention was one of the major strengths of this study. This allowed to avoid the “novelty effect”, and to help families to incorporate the vouchers into their purchase patterns, and therefore in their daily dietary habits. Besides, the absence of significant impact of workshops on food sufficiency status tended to reinforce the association we observed between vouchers and FI. Finally, we used an international validated questionnaire to assess FI [39].

Some limitations should be discussed. First, we faced difficulties in the recruitment process and therefore included much less families than we expected. This might have led to an insufficient power, and therefore to the under-estimation of several associations in our sample. Moreover, we cannot exclude a desirability bias in the intervention group when follow-up questionnaires were administered. Households who received food vouchers over one year were probably more likely to minimise their FI, due to Hawthorne effect [24, 56]. Most parents were born abroad. Given dietary habits are strongly associated with ethnic and cultural backgrounds, we assume that fruits and vegetables consumption or vouchers use could have been different in this particular population [64–67]. Caution is therefore needed regarding the generalization of the results. Although the intervention was randomly assigned, food insufficiency at inclusion was somewhat higher in the intervention group compared to the control group (85.3% vs 70.0%). Despite it was not significant (Fisher exact test, *p* = 0.23, data not tabulated), this difference at inclusion could partly explain the results. Besides, the proportion of families with food insufficiency at follow-up was not statistically significant between the two groups (Fisher exact test, *p* = 0.60, data not tabulated). Finally, we could not explore the impact of vouchers on FI using the complete 18-items FI module, since this latter was not used for follow-up questionnaire. But the 4-items food sufficiency indicator has been shown to fairly estimate FI prevalence when combining the three questions on qualitative and quantitative food insufficiency [43].

## Conclusions

According to previous results of similar works performed in other developed countries, and in the scope of the French national Nutritional health program, results of this pilot study suggest that the allotment of fruits and vegetable vouchers over a one-year period might be a useful lever to alleviate the food insecurity of low-income households who have no access to food aid programs. Beyond improving access to fruits and vegetables, the priority of public health policies should always remain access to sufficient food.

## Abbreviations

FAO: Food and Agriculture Organization
FI: Food Insecurity
FLAM: Fruits and vegetables at home (“*Fruits et légumes à la maison”)*
FSI: Food Sufficiency Indicator
USDA HFSM: Household food Security Module by the US department of Agriculture
